# COVID-19 vaccination in relation to immune response and physical activity levels

**DOI:** 10.3389/fpubh.2026.1839099

**Published:** 2026-09-08

**Authors:** Ioanna Voulgaridi, Petros C. Dinas, Zacharoula Bogogiannidou, Katerina Dadouli, Athanasios G. Lianos, Nikolas Papageorgiou, Dimitrios Georgiou, Nikolaos Zigas, Eleni Bouloumpasi, Evangelia Syrakouli, Argyro Syrakouli, Styliani Sarrou, Maria Tseroni, Fani Kalala, Varvara A. Mouchtouri, Matthaios Speletas, Yiannis Koutedakis, Christos Hadjichristodoulou

**Affiliations:** 1 Laboratory of Hygiene and Epidemiology, Faculty of Medicine, University of Thessaly, Larissa, Greece; 2 FAME Laboratory, Department of Physical Education and Sport Science, University of Thessaly, Trikala, Greece; 3 Department of Immunology and Histocompatibility, Faculty of Medicine, University of Thessaly, Larissa, Greece; 4 Nursing Department, National and Kapodistrian University of Athens, Athens, Greece

**Keywords:** antibodies, COVID-19, exercise, immunization, physical activity, SARS-CoV-2, vaccines

## Abstract

**Introduction:**

The study aimed to determine the antibody response associated with physical activity (PA) levels and participants’ individual characteristics following coronavirus disease 2019 (COVID-19) vaccination.

**Methods:**

The study population included adults vaccinated with either mRNA, adenovirus vector-based, or protein-based adjuvanted vaccines. The sample was derived from participants in a larger cohort study, whose blood samples were collected on days 21, 42, 90, and 180 following COVID-19 vaccination. We tested for immunoglobulin G (IgG) and immunoglobulin A (IgA) anti-severe acute respiratory syndrome coronavirus 2 (SARS-CoV-2) antibody responses, excluding samples collected on day 180, which were analyzed only for IgG antibody titers. Physical Activity (PA) levels were estimated using the short-form International PA Questionnaire (IPAQ), which measures usual weekly physical activity.

**Results:**

Overall, 641 individuals participated in the study. Seropositivity for IgG antibodies was significantly higher among participants with moderate or high PA levels at 21 days (92.6 and 97.0%, respectively) than among those in the low exercise intensity group (70.4%) (*p* < 0.001). Increasing age was associated with reduced IgG antibody production across all time points (*p* < 0.001). The presence of at least one comorbidity was also linked to lower IgG and IgA titers at all time points (IgG: *p* < 0.05; IgA: day 21, *p* < 0.001; days 42 and 90, *p* > 0.05). Vaccine type, COVID-19 history, and PA levels significantly affected IgG and IgA antibody responses at specific time points.

**Conclusion:**

Encouraging moderate-to-high levels of exercise regimens before immunization could enhance vaccine efficacy, particularly among groups at risk of poor immune responses, such as older adults and those with chronic diseases.

## 1 Introduction

The World Health Organization (WHO) estimated that severe acute respiratory syndrome coronavirus 2 (SARS-CoV-2) infections had resulted in more than 7 million deaths worldwide as of March 2026 (1). The coronavirus disease 2019 (COVID-19) pandemic disrupted daily life worldwide, with significant impacts on public health, including mental health (2). The pandemic triggered a cascade of unfavorable events, affecting not only individuals’ health but also their everyday living conditions at personal, social, professional, and economic levels, with global gross domestic product (GDP) declining by 3.4% (3, 4). Human populations had to respond to the pandemic. As no medicines were initially available and antibiotics were ineffective against COVID-19 (5), health organizations relied on countermeasures and vaccinations to control the spread of the virus.

During the pandemic, several vaccines were developed, including mRNA vaccines, adenovirus vector-based vaccines, and protein-based adjuvanted vaccines. In addition to immunization, the WHO also recommended a range of measures to protect the public against COVID-19, including non-pharmaceutical protective measures, a healthy diet, and adequate physical activity (PA) (6).

Higher levels of PA have been associated with enhanced immune responses following vaccination (7). In addition, recent evidence indicates that COVID-19 vaccines may influence broader health-related outcomes beyond immunogenicity, including physical performance and quality of life, which may indirectly interact with immune function (2). Engaging in PA is negatively associated with the risk of community-acquired infectious disease (hazard ratio 0.69, 95% CI 0.61–0.78), representing a 31% lower risk, along with increased immunoglobulin A (IgA) responses to vaccination and higher CD4 T-cell concentrations following vaccination (8).

Chronic aerobic exercise and high PA levels are associated with higher influenza antibody titres after vaccination, compared to individuals who do not engage in exercise or PA (9). Therefore, we hypothesized that PA levels could also play a role in antibody responses following COVID-19 vaccination. The existing literature presents conflicting evidence regarding the direction of the association between PA and post-vaccination antibody responses, with some studies reporting a positive association, whereas others have reported an inverse association (2, 10–13). We aimed to determine whether the titer of antibodies was associated with PA levels and participants’ individual characteristics—such as age, sex, body mass index (BMI), comorbidities, and type of vaccination—following COVID-19 vaccination.

## 2 Materials and methods

### 2.1 Study design

We conducted a retrospective cohort study using telephone interviews to assess participants’ PA levels during the period when they received COVID-19 vaccination.

### 2.2 Study population

Participants were adults who received either mRNA vaccines (BNT162b2: Comirnaty®, BioNTech/Pfizer, Mainz, Germany), adenovirus vector-based vaccines (ChAdOx1 nCoV-19: AZD1222, Oxford/AstraZeneca, University of Oxford, UK or Ad26. COV2. S: Janssen Biotech, Inc., Janssen Pharmaceutical Companies, Johnson and Johnson, New Brunswick, NJ, United States), or the protein-based adjuvanted vaccine Nuvaxovid (NVX-CoV2373, Novavax). The sample included participants from the parent cohort study (*N* = 821) who agreed to participate.

In the parent cohort study, blood samples were collected from participants at specific time points following vaccination, namely on days 21, 42, 90, and 180 after the first dose. Vaccinations were administered from 12 December 2020 to 13 July 2022 at vaccination centers located within the Regional Unit of Larissa, Greece.

### 2.3 Data collection

Participants were interviewed from March to May 2023 to record PA levels, weight, and height. PA was assessed based on participants’ reports of a typical week prior to receiving the first vaccination dose.

At enrollment in the parent cohort study, demographic and medical history data—including a history of COVID-19, the type of COVID-19 vaccine received, and adverse reactions to vaccination—were collected using a questionnaire. Further details on data collection have been previously described (14, 15). A positive history of COVID-19 was defined as a self-reported positive polymerase chain reaction (PCR) result from a nasopharyngeal swab. Furthermore, participants were considered to have a positive history of COVID-19 if their serum samples showed positive titers for anti-N immunoglobulin G (IgG) antibodies against SARS-CoV-2.

### 2.4 Assessments of physical activity (PA)

To assess PA levels, we used the short-form International Physical Activity Questionnaire (IPAQ), which assesses physical activity during a usual week and has been validated in Greek populations (16, 17) and used in clinical populations (18, 19). IPAQ data were calculated and expressed as metabolic equivalent of task (MET)-minutes per week and categorized into low, moderate, and high PA levels according to the Guidelines for Data Processing and Analysis of the IPAQ, November 2005 (20–22). Participants who did not meet the criteria for moderate or high levels of PA were classified as having a “low” PA level. Participants classified as having a “moderate” PA level met at least one of the following criteria: (a) 3 or more days of vigorous-intensity activity for at least 20 min per day; (b) 5 or more days of moderate-intensity activity and/or walking for at least 30 min per day; or (c) 5 or more days of any combination of walking, moderate-intensity, or vigorous-intensity activities, achieving a minimum total PA of 600 MET-minutes per week. To be classified as having To meet the criteria for a “high” PA level, participants had to meet one of the following criteria: (a) vigorous-intensity activity on at least 3 days per week, achieving a minimum total physical activity of 1,500 MET-minutes per week; or (b) any combination of walking, moderate-intensity, or vigorous-intensity activities on 7 or more days per week, achieving a minimum total PA of 3,000 MET-minutes per week. MET calculation was performed as follows:

- Low (Walking) = 3.3 METs*minutes of walking*days of activity.
- Moderate intensity = 4 METs *minutes of walking*days of activity.
- High intensity = 8 METs*minutes of walking*days of activity.
- Total MET-minutes per week = Walking (METs*minutes*days) + Moderate (METs*minutes*days) + Vigorous (METs*minutes*days).

Activity durations of <10 min were not included, and durations >180 min were truncated (17).

### 2.5 Laboratory analyses

All samples were tested for IgG and IgA anti-SARS-CoV-2 antibodies, except for samples collected on day 180, which were analyzed only for IgG antibody titers. The IgA response was not assessed for participants vaccinated with the protein-based adjuvanted vaccine Nuvaxovid due to limited laboratory capacity. The detection of IgG and IgA antibodies targeting the SARS-CoV-2 spike (S1) protein in serum was performed using commercially available kits. The presence of anti-SARS-CoV-2 anti-S IgG antibodies was measured using a chemiluminescent microparticle immunoassay (CMIA) with the Abbott SARS-CoV-2 IgG II assay on the ARCHITECT i2000SR immunoassay analyzer (Abbott, Abbott Park, IL, United States). The detection of anti-SARS-CoV-2 anti-S IgA antibodies was performed using the SERION ELISA agile SARS-CoV-2 IgA ESR400A kit (Serion, Würzburg, Germany), according to the manufacturer’s instructions. The cutoff for a positive anti-SARS-CoV-2 IgG result was defined as a concentration of 50 AU/mL, while the cutoff for IgA was 10 U/mL. Finally, the presence of anti-nucleocapsid (anti-N) anti-SARS-CoV-2 IgG antibodies, which were used as markers of SARS-CoV-2 infection, was assessed using the Abbott SARS-CoV-2 IgG assay. The aforementioned methods have been described previously (23).

### 2.6 Statistical analyses

Categorical variables are presented as frequencies and percentages. To evaluate the potential impact of missing data due to participant dropout, sensitivity analyses were conducted using both complete-case and available-case analyses for IgG and IgA measurements. Continuous variables are presented as medians and interquartile ranges (IQRs). Continuous variables were analyzed using the Mann–Whitney U test and Spearman’s rank correlation coefficient when the normal distribution of variables was violated. Normality was assessed using the Shapiro–Wilk normality test. To account for potential confounding, variables known or hypothesized to be associated with antibody responses—including age, sex, BMI, vaccine type, comorbidities, and COVID-19 history—were assessed in univariate analyses and subsequently included in multivariable models based on clinical relevance and prior literature (14, 15, 24).

Multivariable linear regression models were used to identify independent predictors of antibody levels. All models were adjusted for age, sex, BMI, vaccine type, comorbidities, categorical score, and COVID-19 history. To further investigate the role of prior SARS-CoV-2 infection, we also conducted multivariable analyses after excluding participants with a history of COVID-19. Vaccine type was categorized as mRNA versus non-mRNA, comorbidities were treated as a binary variable (≥1 vs. none), and the categorical score was treated as a continuous variable. Antibody levels were analyzed as continuous outcomes. Regression coefficients (β) with 95% confidence intervals (CIs) are reported.

Analyses were performed using the R programming language (version 4.2.2; R Core Team, R: A Language and Environment for Statistical Computing, R Foundation for Statistical Computing, Vienna, Austria; available at http://www.R-project.org/). The statistical significance level was set at a *p*-value of <0.05.

## 3 Results

### 3.1 Participants’ demographic data and medical history

A total of 641 individuals (response rate: 78%) participated, including 348 (54.3%) female individuals. The mean age was 60 (±18,9) years, and the mean BMI was 26,55 (±4,95). Moreover, 404 (63%) participants reported at least one diagnosed underlying disease, and 93 (14.5%) had a positive history of COVID-19 prior to vaccination. The majority of participants received the BNT162b2 vaccine (434, 67.7%), followed by ChAdOx1 nCoV-19 (124, 19.3%), Ad26. COV2. S (66, 10.3%), and NVX-CoV2373 (17, 2.7%). All participants received the recommended vaccination doses based on commercially available guidelines and the national immunization strategy. Overall, 249 participants (38.8%) reported low PA levels, 222 (34.6%) reported moderate PA levels, and 170 (26.5%) reported high PA levels.

The sensitivity analysis comparing complete-case and available-case participants in the IgG analysis showed that the two groups differed in terms of age, vaccine type, comorbidities, and PA level (Table 1). Participants included in the complete-case analysis had a higher median age than those in the available-case analysis (58.0 vs. 53.5 years, *p* = 0.001), a lower proportion reporting moderate and high PA levels (32.9% vs. 38.3, and 22.8% vs. 34.5%; *p* < 0.001), and a higher prevalence of comorbidities (66.9% vs. 54.9%; *p* = 0.004). In addition, a greater proportion of complete-case participants had received an mRNA vaccine compared to available-case participants (78.4% vs. 45.1%).

**Table 1:** Baseline characteristics and sensitivity analysis results for IgG measurements.

Characteristics	Available-case (*N* = 206)	Complete-case (*N* = 435)	*p*-value
Sex			
Female	101 (49.0%)	247 (56.8%)	0.079
Male	105 (51.0%)	188 (43.2%)	
Age			
Median [IQR]	53.5 [23.0]	58.0 [29.0]	0.001
Missing	0 (0%)	1 (0.2%)	
BMI			
Underweight	2 (1.0%)	16 (3.7%)	0.246
Healthy weight	83 (40.3%)	162 (37.2%)	
Overweight	76 (36.9%)	156 (35.9%)	
Obesity	45 (21.8%)	101 (23.2%)	
Vaccine			
mRNA	93 (45.1%)	341 (78.4%)	<0.001
non-mRNA	113 (54.9%)	94 (21.6%)	
Comorbidities			
No	93 (45.1%)	144 (33.1%)	0.004
Yes	113 (54.9%)	291 (66.9%)	
Categorical score			
1	56 (27.2%)	193 (44.4%)	<0.001
2	79 (38.3%)	143 (32.9%)	
3	71 (34.5%)	99 (22.8%)	
COVID-19 history			
No	175 (85.0%)	373 (85.7%)	0.883
Yes	31 (15.0%)	62 (14.3%)	

Participants included in the complete-case analysis for IgA measurements had a higher median age than those in the available-case analysis (58.0 vs. 50.0 years, *p* < 0.001), a lower proportion reporting moderate and high PA levels (32.4% vs. 35.5 and 24.2% vs. 37.1%; *p* = 0.029), and a higher prevalence of comorbidities (65.3% vs. 51.6%; *p* = 0.050; Table 2).

**Table 2:** Baseline characteristics and sensitivity analysis results for IgA measurements.

Characteristics	Available-case (*N* = 62)	Complete-case (*N* = 475)	*p*-value
Sex			
Female	38 (61.3%)	257 (54.1%)	0.350
Male	24 (38.7%)	218 (45.9%)	
Age			
Median [IQR]	50.0 [12.5]	58.0 [29.0]	<0.001
Missing	0 (0%)	1 (0.2%)	
BMI			
Underweight	1 (1.6%)	16 (3.4%)	0.823
Healthy weight	25 (40.3%)	182 (38.3%)	
Overweight	21 (33.9%)	175 (36.8%)	
Obesity	15 (24.2%)	102 (21.5%)	
Vaccine			
mRNA	54 (87.1%)	350 (73.7%)	0.031
non-mRNA	8 (12.9%)	125 (26.3%)	
Comorbidities			
No	30 (48.4%)	165 (34.7%)	0.050
Yes	32 (51.6%)	310 (65.3%)	
Categorical score			
1	17 (27.4%)	206 (43.4%)	0.029
2	22 (35.5%)	154 (32.4%)	
3	23 (37.1%)	115 (24.2%)	
COVID-19 history			
No	57 (91.9%)	407 (85.7%)	0.249
Yes	5 (8.1%)	68 (14.3%)	

### 3.2 Comparison of IgG and IgA seropositivity following vaccination in individuals with different PA levels and vaccine types

**Figure 1:**
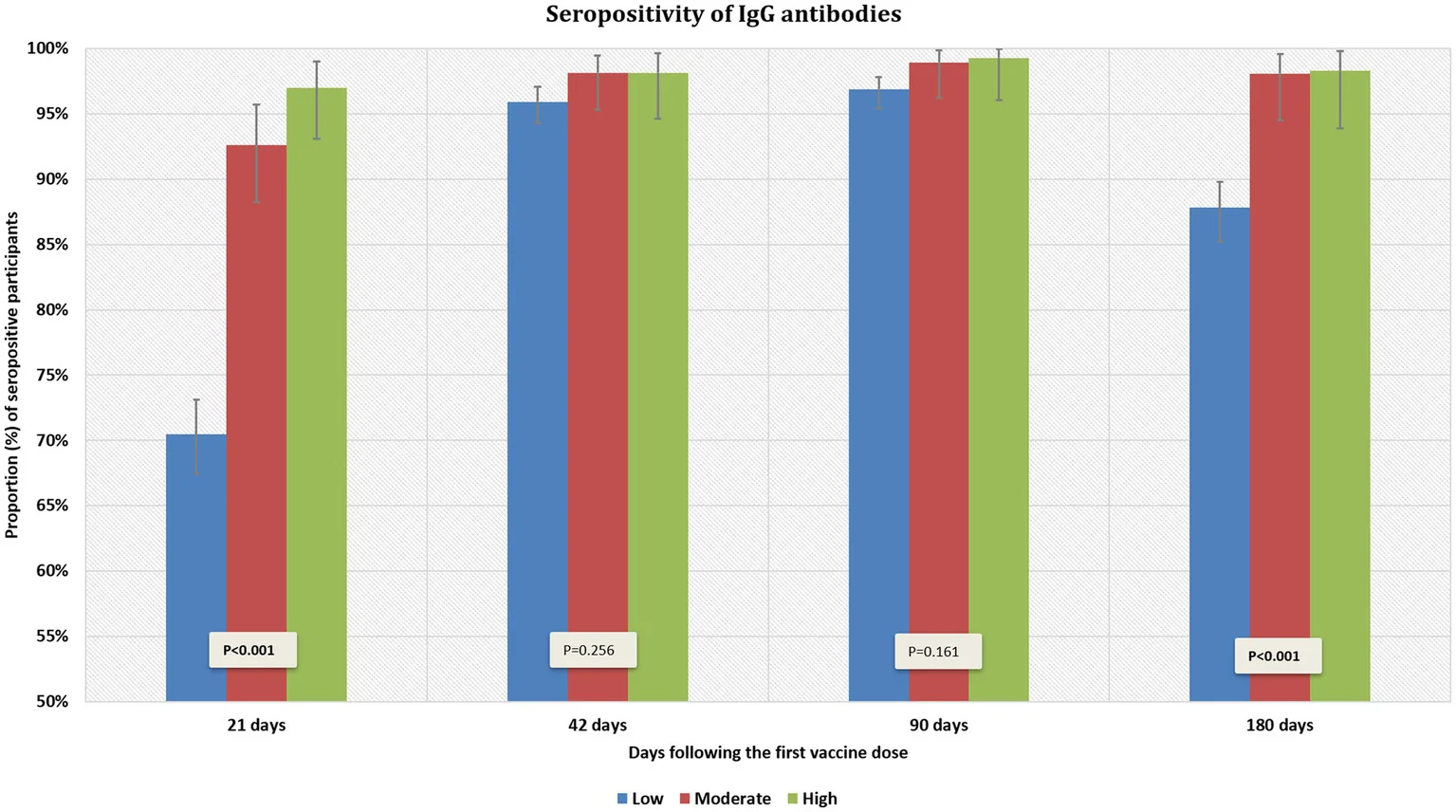
Seropositivity (%) for IgG antibodies at specific time points following the first vaccine dose across three different intensity categories of PA. Corresponding *p*-values are displayed in the figure.

**Figure 2:**
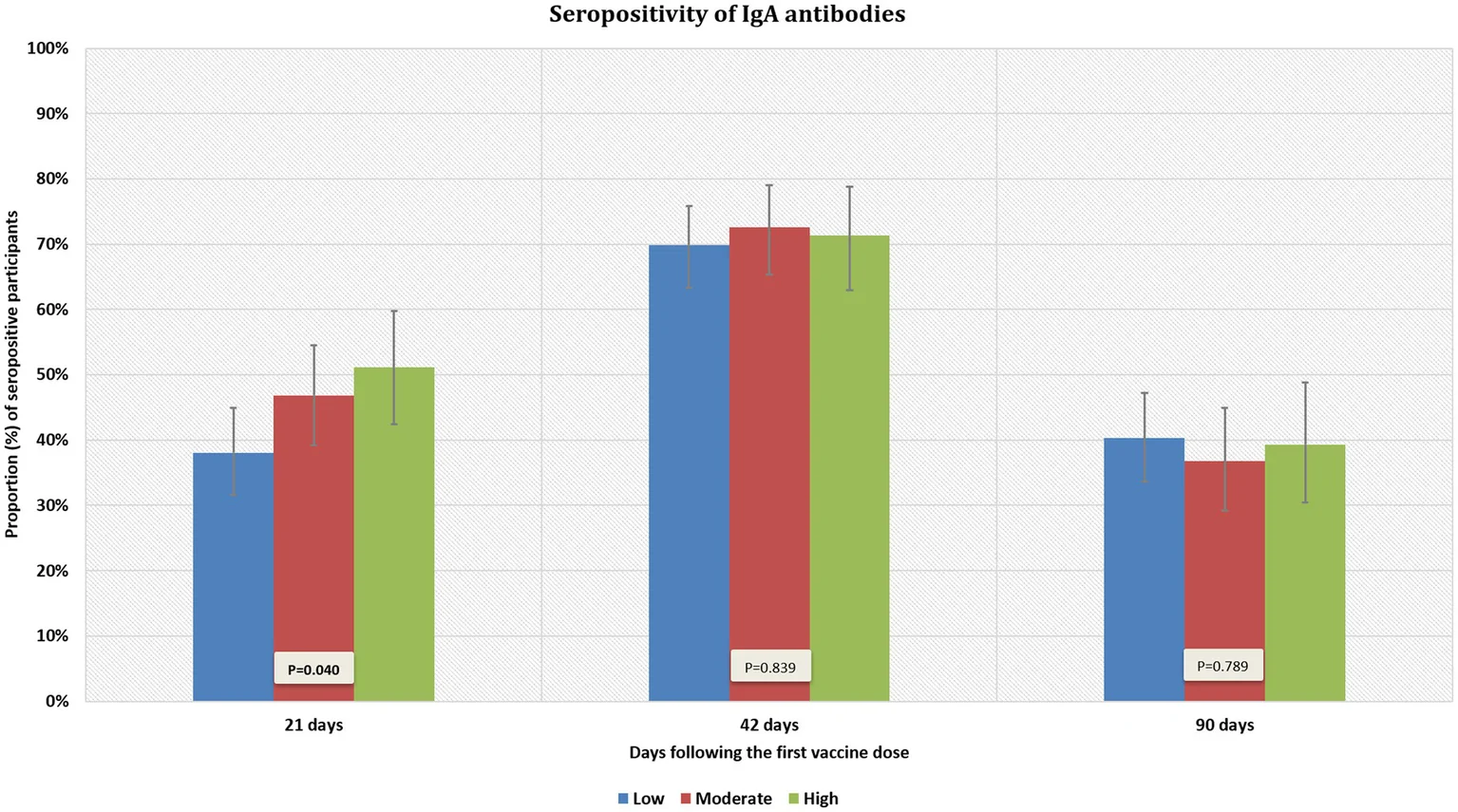
Seropositivity (%) of IgA antibodies at specific time points following the first vaccine dose across three different intensity categories of PA. Corresponding *p*-values are displayed in the figure.

Overall, 629 blood samples were collected on day 21, 620 on day 42, 554 on day 90, and 470 on day 180. Seropositivity for IgG antibodies was associated with PA intensity and was significantly higher among participants with moderate or high PA levels at day 21 (92.6 and 97.0%, respectively) than among those in the low-intensity group (70.4%) (*p* < 0.001; Figure 1). The positivity ratio exceeded 95% for all three groups at days 42 and 90 following the initial vaccination. At day 180, the proportion of participants with positive antibody titers remained statistically significantly higher among individuals in the moderate- and high-intensity PA groups (98.1 and 98.3%, respectively) than among those in the low-intensity group (87.8%) (*p* < 0.001). Despite the low seropositivity of IgA antibodies at day 21, a statistically significant difference was identified among the different PA categories (*p* = 0.040). At days 42 and 90 following the initial vaccination, no statistically significant differences among these categories were observed (Figure 2). Regarding vaccine type, recipients of non-mRNA vaccines exhibited a significantly higher IgG seropositivity rate at 21 days after vaccination than recipients of mRNA vaccines (89.4% vs. 80.6%, *p* = 0.0008). However, at subsequent time points, mRNA vaccines were associated with higher IgG seropositivity rates. For the IgA response, mRNA vaccines induced higher seropositivity rates at all evaluated time points. Detailed results are presented in Supplementary Figures S1, S2.

### 3.3 Univariate analyses of IgG and IgA antibody responses

The results of the univariate analyses for IgG and IgA antibody titers are presented in Tables 3, 4, respectively. Statistically significant differences were observed among vaccine types at all study time points (*p* ≤ 0.004). A history of COVID-19 was associated with statistically significant increases in IgG and IgA antibody levels (*p* < 0.001). Increasing age was associated with reduced IgG antibody production across all time points, demonstrating a statistically significant negative correlation (*p* < 0.001). A similar age-related trend was observed for IgA antibodies; however, the differences were not statistically significant on days 42 and 90 (day 21: *p* < 0.001; day 42: *p* > 0.05; day 90: *p* > 0.05). The presence of at least one comorbidity was also associated with lower IgG and IgA titers (IgG: day 21, *p* < 0.001; day 42, *p* = 0.002; day 90, *p* = 0.003; day 180, *p* < 0.001; IgA: day 21, *p* < 0.001; day 42, *p* > 0.05; day 90, *p* > 0.05). Detailed associations between antibody titers and specific comorbidities are shown in Tables 3, 4. BMI was not found to be a statistically significant factor affecting antibody responses.

**Table 3:** Univariate analysis of anti-SARS-CoV-2 IgG antibodies.

Characteristics		21 days (*N* = 629)		42 days (*N* = 620)		90 days (*N* = 554)		180 days (*N* = 470)	
		N, antibody titer, median AU/ml (IQR)	Sig.	N, antibody titer, median AU/ml (IQR)	Sig.	N, antibody titer, median AU/ml (IQR)	Sig.	N, antibody titer, median AU/ml (IQR)	Sig.
Sex	Male	288, 363 (1170)	0.950	282, 3,830 (12400)	**0.013**	246, 1730 (3870)	0.807	204, 550 (1850)	0.641
	Female	341, 439 (897)		338, 6,170 (12800)		308, 1930 (3160)		266, 597 (966)	
Age		Rho = −0.384	**<0.001**	Rho = −0.204	**<0.001**	Rho = −0.192	**<0.001**	Rho = −0.326	**<0.001**
ΒΜΙ		Rho = 0.003	0.931	Rho = −0.041	0.298	Rho = −0.018	0.669	Rho = 0.003	0.957
Type of vaccine	BNT162b2	428, 440 (1100)	**<0.001**	429, 9,320 (13900)	**<0.001**	403, 2,150 (3480)	**<0.001**	354, 639 (1110)	**0.002**
	ChAdOx1 nCoV-19	119, 240 (449)		109, 281 (552)		71, 845 (1610)		68, 320 (869)	
	Ad26. COV2. S	65, 335 (1180)		65, 367 (1160)		65, 521 (1990)		44, 310 (2510)	
	NVX-CoV2373	17, 9,900 (14100)		17, 6,290 (15300)		15, 3,620 (7550)		4, 7,720 (11000)	
Comorbidities	No	231, 617 (1150)	**<0.001**	228, 8,870 (14800)	**0.002**	204, 2,380 (3500)	**0.003**	164, 839 (1400)	**<0.001**
	Yes (> = 1)	398, 260 (775)		392, 4,440 (11100)		350, 1,580 (3180)		306, 464 (964)	
Comorbidities >2	<=1	484, 475 (932)	**<0.001**	476, 5,880 (13300)	0.380	423, 1970 (3430)	**0.044**	354, 641 (1150)	**0.004**
	Yes (> = 2)	145, 155 (566)		144, 4,410 (11700)		131, 1,300 (3180)		116, 347 (991)	
Autoimmune/autoinflammatory disorders	No	594, 419 (965)	0.293	585, 5,310 (13300)	0.989	522, 1830 (3470)	0.708	438, 592 (1150)	0.826
	Yes	35, 293 (784)		35, 5,410 (10000)		32, 1990 (3060)		32, 491 (1590)	
Dementia/psychiatric/neurological disease	No	476, 446 (903)	**0.003**	468, 5,190 (12900)	0.080	410, 1800 (3210)	0.809	341, 626 (1100)	0.078
	Yes	153, 216 (1180)		152, 5,570 (14300)		144, 1830 (4290)		139, 455 (1300)	
Respiratory disorders	No	601, 397 (939)	0.626	593, 5,280 (12600)	0.118	530, 1800 (3390)	0.351	448, 590 (1180)	0.587
	Yes	28, 592 (4400)		27, 6,490 (24200)		24, 3,290 (6310)		22, 567 (699)	
Cardiovascular disorders	No	343, 566 (1120)	**<0.001**	340, 7,680 (14700)	**<0.001**	302, 2,180 (3510)	**<0.001**	252, 819 (1400)	**<0.001**
	Yes	286, 208 (638)		280, 2,720 (10200)		252, 1,140 (2760)		218, 374 (800)	
Metabolic disorders and endocrinopathies	No	482,459 (973)	**<0.001**	474, 5,700 (13400)	0.176	423, 1960 (3490)	0.113	358, 641 (1170)	**0.011**
	Yes	147, 223 (693)		146, 4,540 (9860)		131, 1,320 (2830)		121, 388 (939)	
Urogenital disorders	No	597, 423 (958)	**<0.001**	587, 5,620 (13200)	**0.018**	526, 1860 (3460)	0.305	442, 605 (1210)	**0.043**
	Yes	32, 108 (196)		33, 1,110 (5180)		28, 1,040 (2760)		28, 318 (692)	
Gastrointestinal disorders	No	611, 416 (949)	0.173	603, 5,470 (13100)	0.569	538, 1860 (3440)	0.471	454, 597 (1170)	0.249
	Yes	18, 176 (751)		17, 2,340 (6330)		16, 907 (3570)		16, 391 (1070)	
Malignancies	No	604, 409 (946)	0.984	595, 5,440 (12900)	0.866	531, 1790 (3380)	0.664	450, 583 (1140)	0.136
	Yes	25, 295 (1270)		25, 2,720 (15300)		23, 1970 (13300)		20, 976 (11300)	
Hematological disorders	No	595, 416 (941)	**0.014**	587, 5,620 (13200)	0.262	523, 1890 (3400)	**0.019**	441, 599 (1200)	**0.032**
	Yes	34, 111 (865)		33, 1,570 (5440)		31, 704 (2610)		29, 274 (797)	
Other	No	585, 417 (958)	**0.011**	574, 5,490 (13200)	0.488	514, 1900 (3450)	0.440	437, 593 (1210)	0.279
	Yes	44, 152 (575)		46, 3,110 (11000)		40, 1,310 (3380)		33, 351 (956)	
Categorical score	Low	247, 199 (655)	**<0.001**	245, 4,140 (10400)	0.098	226, 1,500 (2900)	**<0.001**	197, 378 (938)	**<0.001**
	Moderate	216, 510 (1140)		214, 7,140 (15000)		189, 2,320 (3510)		157, 820 (1540)	
	High	166, 551 (1120)		161, 5,710 (13700)		139, 1710 (3520)		116, 652 (1180)	
COVID-19 history	No	536, 294 (625)	**<0.001**	526, 4,440 (11200)	**<0.001**	463, 1,590 (2520)	**<0.001**	395, 476 (808)	**<0.001**
	Yes	93, 13,100 (23600)		94, 16,600 (34400)		91, 7,960 (18300)		75, 4,350 (11200)	

IQR, Interquartile range; BMI, Body mass index.

**Table 4:** Univariate analysis of anti-SARS-CoV-2 IgA antibodies.

Multiple regression		21 days (*N* = 528)		42 days (*N* = 530)		90 days (*N* = 483)	
		N, antibody titer, median AU/ml (IQR)	Sig.	N, antibody titer, median AU/ml (IQR)	Sig.	N, antibody titer, median AU/ml (IQR)	Sig.
Sex	Male	239, 10.6 (23.1)	**0.001**	239, 19.5 (24.2)	0.469	221, 8.8 (17.8)	**0.043**
	Female	289, 6.8 (13.6)		291, 19.0 (20.9)		262, 6.8 (9.5)	
Age		Rho = −0.252	**<0.001**	Rho = −0.071	0.104	Rho = −0.037	0.415
ΒΜΙ		Rho = −0.072	0.097	Rho = −0.048	0.271	Rho = −0.060	0.190
Type of vaccine	BNT162b2	396, 8.5 (16.2)	**0.004**	398, 23.0 (17.8)	**<0.001**	357, 8.3 (12.1)	**<0.001**
	ChAdOx1 nCoV-19	69, 4.4 (10.4)		68, 2.6 (7.6)		65, 3.6 (9.9)	
	Ad26. COV2. S	63, 10.3 (15.5)		64, 6.5 (19.1)		61, 5.2 (20.3)	
	NVX-CoV2373	-		-		-	
Comorbidities	No	191, 11.3 (13.8)	**<0.001**	194, 20.4 (18.7)	0.407	168, 8.5 (12.4)	0.419
	Yes (> = 1)	337, 6.3 (16.5)		336, 18.3 (24.0)		315, 7.0 (12.3)	
Comorbidities >2	<=1	405, 8.8 (15.1)	**0.010**	407, 19.0 (22.5)	0.131	360, 7.4 (12.0)	0.064
	Yes (> = 2)	123, 5.3 (16.0)		123, 19.6 (21.3)		123, 8.3 (15.3)	
Autoimmune/autoinflammatory disorders	No	497, 8.4 (16.1)	0.076	499, 19.5 (22.6)	0.734	453, 7.8 (12.5)	0.916
	Yes	31, 6.6 (16.8)		31, 15.2 (24.3)		30, 5.4 (13.5)	
Dementia/psychiatric/neurological disease	No	394, 8.6 (14.2)	0.273	395, 18.8 (22.9)	**0.023**	346, 7.3 (10.1)	**0.005**
	Yes	134, 6.3 (25.5)		135, 22.1 (24.1)		137, 8.5 (21.0)	
Respiratory disorders	No	506, 8.3 (16.2)	0.849	508, 18.9 (23.0)	0.380	464, 7.6 (12.9)	0.497
	Yes	22, 5.2 (10.5)		22, 25.5 (15.2)		19, 9.0 (8.6)	
Cardiovascular disorders	No	292, 10.3 (14.8)	**<0.001**	295, 20.3 (19.3)	0.110	257, 8.1 (11.7)	0.393
	Yes	236, 5.4 (14.9)		235, 17.4 (25.8)		226, 7.1 (13.8)	
Metabolic disorders and endocrinopathies	No	402, 8.9 (15.9)	**0.013**	405, 19.9 (22.1)	0.572	369, 7.9 (12.2)	0.735
	Yes	126, 5.5 (15.2)		125, 16.5 (23.7)		114, 6.5 (13.0)	
Urogenital disorders	No	500, 8.3 (15.7)	0.571	502, 19.1 (22.7)	0.398	455, 7.7 (12.5)	0.615
	Yes	28, 7.9 (22.2)		28, 23.9 (21.3)		28, 7.9 (16.5)	
Gastrointestinal disorders	No	514, 8.3 (15.8)	0.703	516, 19.5 (22.4)	0.252	467, 7.5 (12.6)	0.692
	Yes	14, 6.6 (23.1)		14, 10.9 (27.4)		16, 10.6 (17.1)	
Malignancies	No	505, 8.3 (16.2)	0.869	507, 19.5 (22.2)	0.692	462, 7.8 (12.3)	0.913
	Yes	23, 7.1 (14.9)		23, 16.6 (28.8)		21, 5.6 (26.5)	
Hematological disorders	No	500, 8.4 (15.9)	0.109	502, 19.5 (22.7)	0.722	454, 7.8 (12.8)	0.996
	Yes	28, 4.5 (17.7)		28, 18.4 (21.8)		29, 6.6 (10.9)	
Other	No	489, 8.3 (16.0)	0.538	490, 19.1 (23.0)	0.880	445, 7.5 (13.0)	0.721
	Yes	39, 7.1 (17.5)		40, 24.0 (19.4)		38, 8.7 (9.1)	
Categorical score	Low	218, 6.7 (15.1)	**0.002**	219, 18.8 (23.9)	0.975	211, 7.1 (13.0)	0.992
	Moderate	175, 9.5 (15.8)		175, 19.5 (21.5)		155, 7.0 (12.7)	
	High	135, 10.7 (16.2)		136, 20.7 (21.9)		117, 8.5 (11.1)	
COVID-19 history	No	456, 6.8 (11.6)	**<0.001**	456, 16.6 (20.9)	**<0.001**	409, 5.9 (9.3)	**<0.001**
	Yes	72, 36.3 (14.6)		74, 37.0 (12.5)		74, 30.0 (20.2)	

IQR, Interquartile range; BMI, Body mass index. Bold values indicate statistically significant results.

When participants were categorized into three groups based on PA levels, a statistically significant association was observed between PA and IgG antibody levels on days 21, 42, 90, and 180 following the initial vaccination (*p* < 0.001). Individuals in the low-intensity PA group consistently showed lower IgG levels compared to those in the moderate- and high-intensity groups (Table 3). For IgA antibodies, a similar effect of PA was observed on day 21 (*p* = 0.002; Table 4). However, no statistically significant differences were found on days 42 and 90 (day 42: *p* = 0.975; day 90: *p* = 0.992).

### 3.4 Multivariate analysis of IgG and IgA antibody responses

Multivariate analysis indicated that vaccine type, a history of COVID-19, and PA levels significantly affected both IgG and IgA antibody responses. Participants vaccinated with mRNA vaccines exhibited significantly higher IgG titers compared to those who received non-mRNA vaccines on days 21, 42, and 90 (*p* = 0.002, *p* < 0.001, and *p* < 0.001, respectively). Similarly, mRNA vaccination also led to significantly higher IgA levels on days 42 and 90 (day 21: *p* = 0.053; day 42: *p* < 0.001; day 90: *p* < 0.001; Tables 5, 6). A history of COVID-19 prior to vaccination was associated with higher antibody levels at all time points for both IgG and IgA (*p* < 0.001). PA intensity was positively associated with antibody responses: Individuals with higher PA levels appeared to have elevated IgG and IgA titers. This association was statistically significant for IgG on day 42 (*p* = 0.021) and for IgA on days 21 and 42 (*p* = 0.047 and *p* = 0.048, respectively); however, the IgA difference was not statistically significant on day 90 (*p* = 0.281; Tables 5, 6).

**Table 5:** Multivariate analysis of anti-SARS-CoV-2 IgG antibodies.

Multiple regression		21 days (*N* = 628)		42 days (*N* = 619)		90 days (*N* = 553)		180 days (*N* = 469)	
		Coeff with 95% CI	Sig.	Coeff with 95% CI	Sig.	Coeff with 95% CI	Sig.	Coeff with 95% CI	Sig.
Age		37 (−7–82)	0.102	−25 (−94–44)	0.469	36 (−23–96)	0.233	14 (−37–64)	0.595
ΒΜΙ		92 (−37–220)	0.163	195 (−2–391)	0.052	127 (−38–293)	0.131	116 (−23–255)	0.101
Sex	Male/Female	−554 (−733 – −840)	0.398	117 (−1853–2086)	0.907	790 (−896–2,476)	0.358	242 (−1,193–1,678)	0.740
Vaccine	Non- mRNA/mRNA	−2,258 (−3,677 – −839)	**0.002**	−13,938 (−16,123 – −11,754)	**<0.001**	−3,468 (−5,406 – −1,530)	**<0.001**	−295 (−1975–1,386)	0.730
Comorbidities (> = 1 vs. 0)		384 (−1,297–2064)	0.654	−146 (−2,733–2,441)	0.912	326 (−1909–2,560)	0.775	−367 (−2,285–1,551)	0.707
Categorical score	Continuous variable	151 (−779–1,082)	0.749	1,673 (248–3,099)	**0.021**	312 (−914–1,539)	0.617	182 (−851–1,215)	0.729
COVID-19 history		18,972 (17196–20,749)	**<0.001**	20,619 (17920–23,318)	**<0.001**	16,182 (13942–18,421)	**<0.001**	10,943 (9032–12,854)	**<0.001**

Bold values indicate statistically significant results.

**Table 6:** Multivariate analysis of anti-SARS-CoV-2 IgA antibodies.

Multiple regression		21 days (*N* = 527)		42 days (*N* = 529)		90 days (*N* = 482)	
		Coeff with 95% CI	Sig.	Coeff with 95% CI	Sig.	Coeff with 95% CI	Sig.
Age		−0.09 (−0.15 – −0.03)	**0.006**	−0.1 (−0.17 – −0.03)	**0.003**	−0.01 (−0.07–0.05)	0.694
ΒΜΙ		−0.03 (−0.21–0.15)	0.733	0.03 (−0.17–0.22)	0.773	−0.04 (−0.21–0.13)	0.668
Sex	Male/Female	1.96 (0.14–3.78)	**0.035**	0.81 (−1.17–2.78)	0.423	1.2 (−0.57–2.96)	0.184
Vaccine	Non-mRNA/mRNA	−2.1 (−4.23–0.03)	0.053	−15.05 (−17.36 – −12.74)	**<0.001**	−3.73 (−5.78 – −1.67)	**<0.001**
Comorbidities (> = 1 vs. 0)		−0.97 (−3.35–1.41)	0.425	−0.54 (−3.12–2.04)	0.683	−0.5 (−2.88–1.88)	0.681
Categorical score	Continuous variable	1.31 (0.02–2.61)	**0.047**	1.42 (0.01–2.82)	**0.048**	0.71 (−0.58–1.99)	0.281
COVID-19 history		23.23 (20.65–25.82)	**<0.001**	18.04 (15.26–20.82)	**<0.001**	19.95 (17.56–22.34)	**<0.001**

IQR, Interquartile range; BMI, Body mass index. Bold values indicate statistically significant results.

The association between PA and IgG antibody levels at day 42 remained statistically significant after excluding participants with prior COVID-19, supporting an independent relationship between PA level and the IgG response to vaccination (*p* = 0.002). However, no significant association was observed between PA and IgA antibody levels among participants without a history of COVID-19 (Supplementary Tables S1, S2).

## 4 Discussion

Our study reveals a robust positive association between PA intensity and humoral immune responses following COVID-19 vaccination, particularly as evidenced by IgG seroconversion rates. Participants who reported moderate or high levels of PA exhibited significantly higher IgG titers at both early (day 21) and later (days 90 and 180) time points than participants reporting low PA levels. This effect remained significant after adjusting for confounders, such as vaccine type, age, comorbidities, and prior SARS-CoV-2 infection. It is worth noting that our findings may underestimate the positive effect of PA on the antibody response because participants included in the complete-case analysis were generally older, had a higher prevalence of comorbidities, and reported lower levels of moderate- and vigorous-intensity physical activity, all of which are factors associated with a less robust immune response (15). Conversely, a greater proportion of participants in the complete-case analysis received mRNA vaccines, which are associated with higher antibody titres than non-mRNA vaccines (14). Prior research on influenza vaccination suggests that regular moderate-to-high levels of PA may enhance antibody titers (9).

IgG is the most common type of antibody found in blood circulation, accounting for approximately 10–20% of total plasma proteins (25). It is produced during both primary and secondary immune responses, exerting its effector functions by activating the classical complement pathway and engaging Fc receptors on macrophages and other phagocytes (26, 27). We found that PA was significantly associated with higher IgG antibody levels 42 days after vaccination. Similar positive associations between PA and post-vaccination antibody responses have been reported in previous studies (10–12, 28, 29). Notably, a 12-week program of moderate aquatic exercise significantly increased serum IgG concentrations in pre-frail older women, highlighting the potential of regular, structured physical activity to boost humoral immunity in this vulnerable population (28). Moreover, even a single 45-min bout of moderate-intensity walking has been shown to elicit a transient increase in serum immunoglobulins, including IgG, IgA, and IgM, underscoring the acute immunomodulatory effects of exercise (29).

In our study, regarding the IgA response, participants with moderate or high levels of PA had significantly higher antibody titers at day 21 compared to those reporting low PA levels. After adjusting for confounders, the positive association between PA and IgA levels was observed at days 21 and 42, whereas the association was attenuated and no longer statistically significant at day 90. This pattern may be explained by several factors, including the reduced sample size at later time points and the fact that serum IgA responses peak earlier and decline more rapidly than IgG (30). The smaller effect estimate at day 90 could reflect the shorter half-life of IgA (31). However, the lack of statistical significance at day 90 should not necessarily be interpreted as evidence of the absence of an association but rather as a finding that warrants confirmation in larger studies with balanced group sizes and extended follow-up.

In the multivariable analysis restricted to participants without prior SARS-CoV-2 infection, the association between physical activity and the IgG response remained significant, suggesting that the observed effect was independent of hybrid immunity. In contrast, the association with IgA antibody levels was attenuated and no longer reached statistical significance. This may reflect the smaller sample size after excluding previously infected participants, resulting in reduced statistical power. It is also possible that IgA responses are more strongly influenced by prior SARS-CoV-2 infection than IgG responses (32, 33).

IgA is the most abundant antimicrobial protein protecting mucosal surfaces, the region where most infections are initiated (27). Secretory IgA could provide an immunological barrier by neutralizing and preventing viral pathogens. A published systematic review and meta-analysis demonstrated that acute exercise increases IgA levels in trained individuals, but it does not affect its levels in untrained individuals (25). In sedentary individuals, moderate chronic exercise training has been shown to increase secretory IgA levels (34). Vaccination is a major strategy to reduce mortality and morbidity rates for several infectious diseases, including COVID-19 (35, 36). Nonetheless, vaccine efficacy varies between individuals, with particularly low responses found in those with reduced immune function, such as older adults. PA performed near the time of immunization may increase the antibody response to vaccination. Several studies have demonstrated that exercise preceding immunization improved the antibody response (2, 10, 11). In addition, lifestyle interventions, such as engaging in regular outdoor physical activity, could enhance immune responses to mRNA vaccines (12). This is particularly important for individuals at increased risk of severe COVID-19. Another study, however, reported that IgG antibody levels were significantly decreased in the physically active group vaccinated with an mRNA vaccine compared to less active participants, possibly due to interactions between the vaccine and the musculoskeletal system (13). Therefore, these conflicting findings warrant further investigation. High-quality prospective studies with larger and more heterogeneous populations, including recipients of different vaccine types, are needed to better characterize the association between PA and post-vaccination antibody responses. From a public health perspective, these results advocate for the integration of structured PA recommendations into vaccination campaigns. Encouraging moderate-to-high levels of exercise regimens before immunization could enhance vaccine efficacy at the population level, particularly among groups at risk of poor immune responses, such as older adults and individuals with chronic diseases. Future randomized controlled trials should determine the optimal exercise modalities and timing relative to vaccination to maximize immunogenicity.

It is reasonable to assume that the present results may have been influenced by methodological limitations. First, there was a considerable time gap between the period during which participants engaged in PA and the telephone interviews used to assess it. This may have introduced recall bias, potentially leading to an overestimation or underestimation of PA intensity. In addition, the use of convenience sampling may have introduced participation bias, thereby limiting the generalizability of our findings. In our study design, PA was assessed only once and therefore reflects participants’ activity before the first vaccination dose rather than throughout the follow-up period, which may have resulted in misclassification bias. Moreover, individuals with higher PA levels may be inherently healthier or more health-conscious, which could independently influence immune outcomes of PA and introduce a potential healthy-volunteer bias. Although we adjusted for comorbidities, this adjustment was limited to a binary variable. Individuals engaging in higher levels of PA are generally healthier, with fewer or less severe chronic conditions, better immune function, and healthier lifestyle behaviors. Consequently, residual confounding due to incompletely measured health characteristics and lifestyle factors cannot be excluded. This healthy-volunteer bias may have led to an overestimation of the observed protective association between PA and antibody response titers.

Furthermore, no baseline samples were available to compare anti-S antibody titers prior to vaccination with subsequent measurements. In some instances, serum samples were insufficient, allowing for the measurement of IgG antibodies only. Consequently, IgA responses induced by the protein-based adjuvanted vaccine Nuvaxovid were not assessed. Another limitation is that serum SARS-CoV-2 antibody levels represent only a partial correlate of vaccine effectiveness, as protective immunity also involves T-cell responses that are not captured by IgA/IgG measurements. Finally, participant response rates varied across different time points, resulting in differences in the number of collected samples.

The study also has notable strengths. Antibody levels were measured at multiple post-vaccination time points, allowing a more comprehensive assessment of the temporal dynamics of the immune response. Participants received different vaccine types, enabling the evaluation of immune responses across various vaccination platforms. Moreover, our sample included individuals with diverse profiles, allowing for the adjustment for and assessment of potential confounding factors.

## 5 Conclusion

Individuals in the low-intensity PA group consistently showed lower IgG levels than those in the moderate- and high-intensity PA groups, with the difference reaching statistical significance on day 42. These findings suggest that promoting moderate- to high-intensity exercise prior to immunization may enhance vaccine efficacy, particularly among populations at risk of poor immune responses, such as older adults and individuals with chronic diseases.

## Data Availability

The raw data supporting the conclusions of this article will be made available by the authors, without undue reservation.
