# Supplementary material for: COVID-19 vaccination in relation to immune response and physical activity levels
Source: Front Public Health. 2026 Sep 8;14:1839099. doi: 10.3389/fpubh.2026.1839099 (PMC13593892; doi:10.3389/fpubh.2026.1839099)
Supplement: Supplementary file 1 [file Supplementary_file_1.DOCX]

|  | 21 ds | | | 42 ds | | | 90 ds | | | 180 ds | | |
| --- | --- | --- | --- | --- | --- | --- | --- | --- | --- | --- | --- | --- |
| *Predictors* | *Estimates* | *CI* | *p* | *Estimates* | *CI* | *p* | *Estimates* | *CI* | *p* | *Estimates* | *CI* | *p* |
| (Intercept) | 1634.43 | -40.43 – 3309.29 | 0.056 | 16674.63 | 11595.33 – 21753.94 | **<0.001** | 3409.84 | 906.33 – 5913.35 | **0.008** | 1115.00 | -1263.53 – 3493.54 | 0.357 |
| AGE | -6.63 | -22.06 – 8.81 | 0.399 | -115.08 | -163.67 – -66.50 | **<0.001** | -13.56 | -38.64 – 11.53 | 0.289 | 1.45 | -22.07 – 24.98 | 0.903 |
| BMI [Healthy Weight] | -437.47 | -1813.06 – 938.11 | 0.532 | 988.73 | -3139.18 – 5116.63 | 0.638 | 194.08 | -1783.93 – 2172.09 | 0.847 | 53.46 | -1813.72 – 1920.64 | 0.955 |
| BMI [Overweight] | -308.80 | -1687.24 – 1069.64 | 0.660 | 1126.82 | -3014.05 – 5267.70 | 0.593 | 338.41 | -1644.41 – 2321.22 | 0.737 | 45.93 | -1820.95 – 1912.81 | 0.961 |
| BMI [Obesity] | -96.48 | -1502.92 – 1309.96 | 0.893 | 2047.80 | -2179.69 – 6275.29 | 0.342 | 363.08 | -1670.47 – 2396.62 | 0.726 | 727.98 | -1181.62 – 2637.58 | 0.454 |
| SEX [Male] | -372.18 | -804.20 – 59.84 | 0.091 | -1993.14 | -3347.47 – -638.81 | **0.004** | -532.44 | -1221.41 – 156.53 | 0.130 | 285.25 | -362.15 – 932.66 | 0.387 |
| Vaccine binary [non mRNA] | -390.86 | -868.12 – 86.41 | 0.108 | -10509.20 | -12017.97 – -9000.43 | **<0.001** | -1008.29 | -1809.45 – -207.14 | **0.014** | 609.52 | -156.50 – 1375.54 | 0.119 |
| Comorbidities [Yes] | 29.99 | -519.32 – 579.30 | 0.915 | -1535.71 | -3265.94 – 194.53 | 0.082 | -407.91 | -1299.95 – 484.14 | 0.369 | -996.90 | -1847.84 – -145.96 | **0.022** |
| Categorical score [Average] | 223.14 | -303.13 – 749.41 | 0.405 | 2635.98 | 991.32 – 4280.65 | **0.002** | 494.99 | -352.62 – 1342.59 | 0.252 | 59.75 | -735.39 – 854.88 | 0.883 |
| Observations | 523 | | | 515 | | | 455 | | | 394 | | |
| R^2^ / R^2^ adjusted | 0.018 / 0.003 | | | 0.362 / 0.352 | | | 0.039 / 0.021 | | | 0.041 / 0.021 | | |

S. table 1. *Multivariable analysis for IgG antibodies excluding participants with a history of SARS-CoV-2 infection*

S. table 2. *Multivariable analysis for IgA antibodies excluding participants with a history of SARS-CoV-2 infection*

|  | 21 ds | | | 42 ds | | | 90 ds | | |
| --- | --- | --- | --- | --- | --- | --- | --- | --- | --- |
| *Predictors* | *Estimates* | *CI* | *p* | *Estimates* | *CI* | *p* | *Estimates* | *CI* | *p* |
| (Intercept) | 17.02 | 10.34 – 23.70 | **<0.001** | 25.10 | 17.71 – 32.50 | **<0.001** | 9.63 | 3.02 – 16.24 | **0.004** |
| AGE | -0.10 | -0.17 – -0.04 | **0.002** | -0.10 | -0.17 – -0.03 | **0.007** | -0.01 | -0.07 – 0.06 | 0.831 |
| BMI [Healthy Weight] | -1.52 | -6.83 – 3.78 | 0.572 | 1.27 | -4.60 – 7.14 | 0.671 | 0.32 | -4.78 – 5.42 | 0.901 |
| BMI [Overweight] | 0.09 | -5.21 – 5.39 | 0.974 | 2.55 | -3.32 – 8.41 | 0.394 | 1.01 | -4.07 – 6.09 | 0.696 |
| BMI [Obesity] | -1.79 | -7.22 – 3.63 | 0.516 | 1.17 | -4.84 – 7.17 | 0.702 | -0.15 | -5.37 – 5.06 | 0.954 |
| SEX [Male] | 1.58 | -0.22 – 3.38 | 0.084 | 0.17 | -1.82 – 2.16 | 0.867 | 0.52 | -1.23 – 2.26 | 0.561 |
| Vaccine binary [non mRNA] | -3.03 | -5.15 – -0.91 | **0.005** | -17.30 | -19.65 – -14.96 | **<0.001** | -3.68 | -5.72 – -1.64 | **<0.001** |
| Comorbidities [Yes] | -1.26 | -3.57 – 1.04 | 0.282 | -0.13 | -2.68 – 2.42 | 0.921 | -1.15 | -3.45 – 1.16 | 0.328 |
| Categorical score [Average] | 0.94 | -1.26 – 3.14 | 0.401 | 1.71 | -0.73 – 4.15 | 0.168 | 0.17 | -2.00 – 2.35 | 0.874 |
| Observations | 443 | | | 445 | | | 402 | | |
| R^2^ / R^2^ adjusted | 0.092 / 0.075 | | | 0.339 / 0.327 | | | 0.037 / 0.017 | | |

Seropositivity of IgG antibodies

Seropositivity (%)

| Days | mRNA (N=434) | non mRNA (N=207) |
| --- | --- | --- |
| 21 | 80.6 | 89.4 |
| 42 | 97 | 87.9 |
| 90 | 91.2 | 71.5 |
| 180 | 76.5 | 52.7 |

Figure S1. Seropositivity of IgG antibodies stratified by vaccine type.

Seropositivity of IgA antibodies

Seropositivity (%)

| Days | mRNA (N=434) | non mRNA (N=207) |
| --- | --- | --- |
| 21 | 41.9 | 25.1 |
| 42 | 78.6 | 17.4 |
| 90 | 34.6 | 18.4 |

Figure S2. Seropositivity of IgA antibodies stratified by vaccine type.
